# The PIRATE mnemonic: providing a structured approach in the care for intoxicated patients at the emergency department

**DOI:** 10.1186/s12245-024-00606-4

**Published:** 2024-03-01

**Authors:** Nicole Kraaijvanger, Wouter Raven, Trudy van Dijken, Femke Gresnigt

**Affiliations:** 1 Leiden University Medical Center, Albinusdreef 2, Leiden, 2333 ZA the Netherlands; 2 Diakonessenhuis, Bosboomstraat 1, Utrecht, 3582 KE the Netherlands; 3 OLVG hospital, Oosterpark 9, Amsterdam, 1091 AC the Netherlands; 4 Toxicology section of Dutch Society of Emergency Physicians, Utrecht, the Netherlands; 5 Medical toxicology at Dutch Poison Information Center, Utrecht, the Netherlands

**Keywords:** Intoxications, Acute toxicology, Emergency department, Mnemonic

## Abstract

**Background:**

Expertise in toxicology is essential for acute care providers, as intoxicated patients frequently present to Emergency Departments. These patients can be challenging for care providers because they often present with uncertain substance exposure and unknown dose and timing of these exposures.

**Methods:**

The Dutch Society of Emergency Physicians has developed an mnemonic to support treating physicians in a structured approach for the management of (undifferentiated) intoxicated patients.

**Results:**

The PIRATE mnemonic was developed, which includes the following aspects and sequence of care for the intoxicated patient: primary survey, investigation & identification, risk assessment, ADME (comprising pharmacokinetic therapeutic targets: absorption, distribution, metabolism, elimination), therapy and evaluation.

**Conclusion:**

The toxicology section of the Dutch Society of Emergency Physicians developed the PIRATE mnemonic to provide a structured approach in the management of patients presenting with acute intoxications to Emergency Departments. It summarizes the essential steps and priorities required in the care of intoxicated patients. Further, it provides a common strategy for all specialties involved in the care of the acutely intoxicated patient, contributing to developing greater competence in poisoning management.

## Background

Toxicology knowledge is essential for emergency physicians and other acute care providers, as intoxicated patients are often seen at Emergency Departments [1–3]. However, patients may present undifferentiated, it may be unclear what substances they were exposed to and there is a wide variety of agents causing intoxications. Moreover, in addition to somatic aspects, often psychosocial issues come into play in an intoxicated patient, including psychiatric problems, addiction or child neglect/abuse. Because of this complexity, a structured approach to managing intoxicated patients that considers the varied aspects involved in this type of emergency department visit is needed. The PIRATE mnemonic provides a structured approach to the care of acute intoxications, can contribute to confidence in acute patient care and serves as a common strategy between specialties. To the best of our knowledge, no other such mnemonic is in use.

Toxicology is part of most emergency medicine curricula, like the curriculum from the International Federation for Emergency Medicine and the European Society for Emergency Medicine [4, 5]. In the Dutch emergency medicine curriculum, a 2-day toxicology course is part of the mandatory training program [6]. Recently an official toxicology rotation, with a three-month duration, was initiated at one Dutch hospital, but a majority of Dutch emergency trainees are not enrolled in such a program. Also, exposure to acute toxins varies by country and region. This means that the level of emergency medicine training and clinical experience, especially concerning specific intoxications, varies and may be limited. In 2021, an American survey found that a minority of emergency medicine residents felt comfortable with the core toxicology content of their curriculum, even when a toxicology rotation was part of their training and they had access to a board-certified toxicologist [7]. 

## Methods

In 2014, the toxicology section of the Dutch Society of Emergency Physicians, at that time consisting of fifteen emergency physicians with expertise in acute medical toxicology, came together as an expert panel and developed the first version of the PIRATE mnemonic (at that time called PIRAAT, in Dutch). The mnemonic’s various elements were based on the workup of poisoned patients as described in textbooks, such as ‘Tintinalli’s Emergency Medicine Manual’ and ‘Goldfrank’s Toxicologic Emergencies’ [8, 9]. There was a need for a directly applicable, concise, and structured but comprehensive mnemonic encompassing all vital aspects of the care of an acutely intoxicated patient, as this was lacking in the toxicology and emergency medicine literature and practice. This first mnemonic was introduced in 2014 in the toxicology courses for Dutch emergency physicians and registrars in emergency medicine. All courses were evaluated with the participants by discussion after the course and a written evaluation. Feedback on the mnemonic was not specifically requested. In 2018, the mnemonic was revised, where feedback from previous courses was taken into account. It was again discussed with an expert panel, this time consisting of five emergency physicians and two Australian, internationally renowned clinical toxicologists. The changes made were based on practical usability, improving the order and priorities of the different elements. In addition, the mnemonic was translated into the current English version. Since 2022, the PIRATE pocket card is free to download from the Dutch Society of Emergency Physicians website [10]. 

## Results

**Fig. 1:**
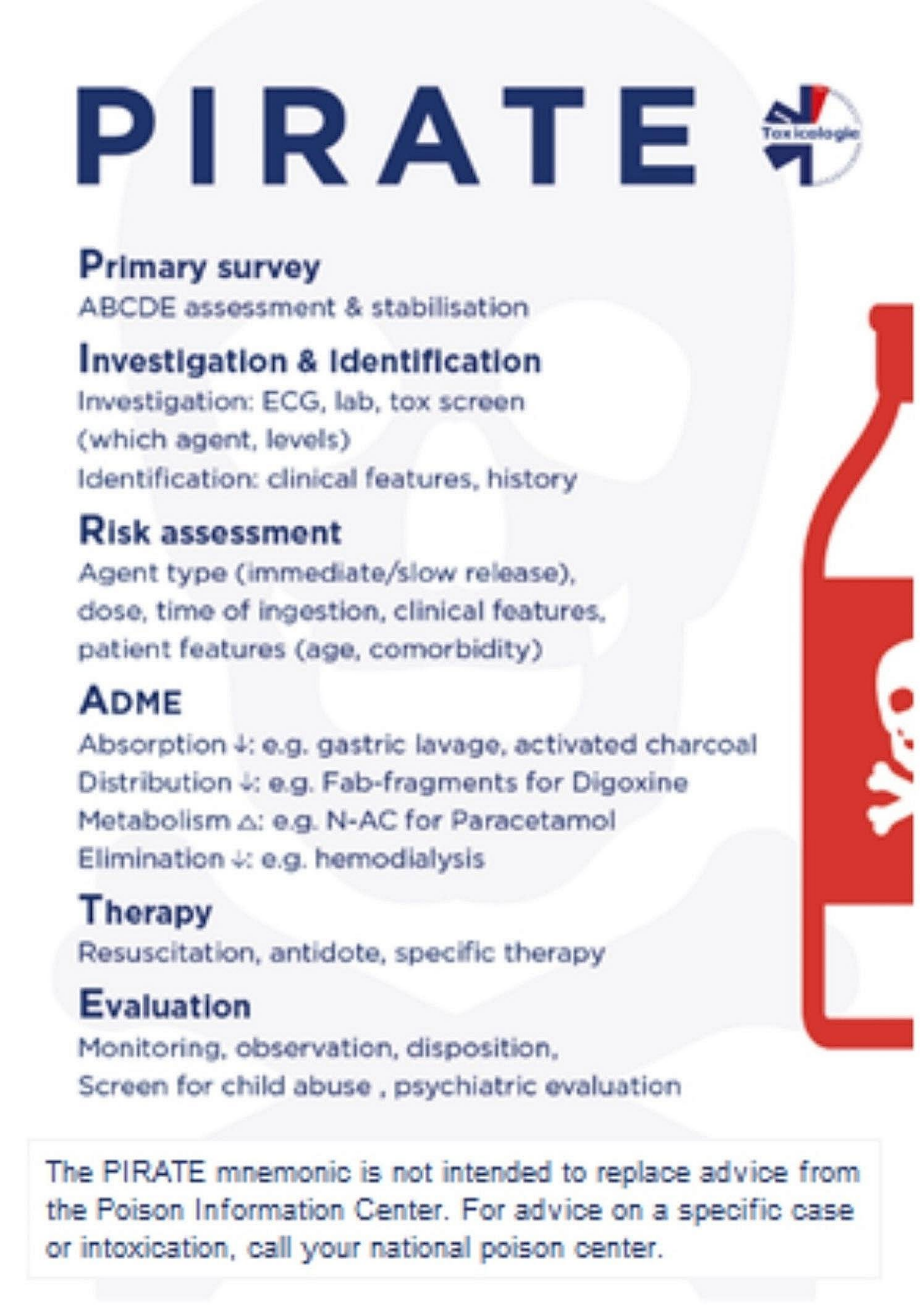
Pocket card as developed by the Dutch Society of Emergency Physicians, presenting the PIRATE mnemonic [10]

The PIRATE mnemonic provides a structured approach for the care of acutely intoxicated patients. Each letter of the PIRATE stands for a different aspect of care. The letters are organized in a sequential order of priorities. The PIRATE mnemonic is available as an easy to take pocket card. (Fig. 1). The PIRATE mnemonic is not intended to replace expert toxicological advice from a clinical toxicologist or Poison Information Center.

### The PIRATE mnemonic

#### P–Primary survey:

Start with resuscitation and stabilization of the patient following the well-known ABCDE approach (airway, breathing, circulation, disability, exposure/environment).

#### I – Investigation & Identification

Investigation: Consider which investigations must be performed to optimize care for the patient. Always perform an ECG. Consider performing laboratory investigations and a toxicology screening or specific drug level(s). Consider a paracetamol level and pregnancy test.

Identification: Use the history, clinical features and (when available) investigation results to classify a certain toxidrome and identify potential causative substances.

#### R – Risk assessment

Combine the clinical assessment and information on the likely agent (or toxidrome) to assess the severity of the intoxication and the possible (forthcoming) effects for this patient. Take into account the (type of) agent, immediate/slow release, dose, route of exposure, time of ingestion, current clinical features and patient features such as age, comorbidity and comedication.

#### A – ADME

This concerns the pharmacokinetic therapeutic targets including: A: Absorption (reduction): consider performing gastric lavage and/or giving single or multiple doses of activated charcoal, or whole bowel irrigation. D: Distribution: consider therapies that interfere with the distribution of an agent, thereby reducing toxicity. For example, Digoxin specific antibodies for Digoxin poisoning. M: Metabolism: consider therapies that interfere with the metabolism of an agent, thereby reducing toxicity. For example, N-acetylcysteine for paracetamol poisoning. E: Elimination (increase): consider therapies that stimulate elimination of an agent. Such as sodium bicarbonate for salicylate poisoning or extracorporeal treatment, for example hemodialysis according to Extracorporeal Treatments in Poisoning (EXTRIP) guidelines [11]. 

#### T - Therapy

Ongoing resuscitation, monitoring and supportive care. In addition, specific therapy, including antidotes, should be considered. Prepare for potential complications.

#### E – Evaluation

Ongoing plan, including patient disposition. Consider indication for continuous monitoring of vital signs and heart rhythm, determine the period and location of observation. In addition, screen the safety of possible children and perform a psychiatric evaluation in case of deliberate self-harm.

Between 2014 and 2018, over 200 emergency physicians and registrars were educated according to the first Dutch PIRAAT mnemonic. Since 2019, over 200 emergency physicians and registrars have been educated according to the English PIRATE mnemonic. Together with national toxicology experts, the initial course for emergency registrars and emergency physicians was transformed into a multidisciplinary toxicology course. Besides the emergency physicians involved in the emergency toxicology course, it is also provided by several consultants from the Dutch Poison Information Center and all members of the Medical Intoxication Forum (Geneeskundig Intoxicatie Forum in Dutch) working group, which is a cooperation of the Dutch Society of Emergency Physicians, the Dutch society of acute internal medicine, the Dutch society of intensive care, the Dutch society of clinical pharmacologists and the Dutch society of hospital pharmacists. At this moment, a total of 199 trainees have followed the multidisciplinary toxicology course and were educated according to the English PIRATE mnemonic. At course evaluations, acute care providers state the PIRATE mnemonic allows them to feel more comfortable and confident in their daily practice. More extensive written feedback is shown in Table 1.

**Table 1:** All spontaneously given written feedback on the PIRATE mnemonic mentioned in course evaluations. (translated to English)

Gained a lot of knowledge, which is highly applicable in practice. Particularly, the PIRATE system.
Maybe some pocket cards to take along, featuring the PIRATE and perhaps some toxidromes would be convenient.
The clear ‘hooks’ provided in the course (PIRATE, toxidromes) make it very applicable in practice.
Consistently revisiting the hooks; PIRATE and toxidromes. This is now really ingrained.
I have received good tools, including the PIRATE, for managing patients with intoxication.
PIRATE is very useful!
For each topic, the basics are reintegrated (PIRATE as a tool and which therapy corresponds to which type of intoxication). Additionally, it’s of great value that you learn to think in patterns and observe what you immediately see in the patient, rather than thinking per medication or per condition.
There is simply a lot of information. This is partly addressed by offering various hooks, like the PIRATE.

## Discussion

The aim of the PIRATE mnemonic is to add structure to the first assessment and management of every acutely poisoned patient. It also supports the emergency care provider by suggesting all toxicological aspects of care. It is important to emphasize that the PIRATE mnemonic and the pocket card are not intended to replace discussion of a case with a clinical toxicologist or Poison Information Center. The mnemonic may however also support the acute care provider with formulating a more specific question for expert consultation and as such get more targeted advice in return. While the PIRATE mnemonic prioritizes the different aspects of care for all intoxicated patients, it does not replace guidelines for specific toxin management.

### Limitations

Although the PIRATE mnemonic is widely supported by Dutch toxicology experts, including consultants from the Dutch Poison Information Center, the mnemonic is expert opinion based and, to date, has not been formally evaluated for its impact on the quality of care.

## Conclusions

The toxicology section of the Dutch Society of Emergency Physicians developed the PIRATE mnemonic, which provides emergency care providers with a structured approach to treating intoxicated patients presenting to emergency departments. Emergency Medicine personnel may be more confident in managing this patient group and all clinicians delivering care to this patient subset are provided with a consistent strategy for patient management.

## Data Availability

Data sharing is not applicable to this article as no datasets were generated or analyzed during the current study.

## Abbreviations

DSEP: Dutch Society of Emergency Physicians
ECTR: Extracorporeal treatment
ED: Emergency Department
